# Adaptive neighborhood triplet loss: enhanced segmentation of dermoscopy datasets by mining pixel information

**DOI:** 10.1007/s11548-024-03241-9

**Published:** 2024-08-02

**Authors:** Mohan Xu, Lena Wiese

**Affiliations:** 1 Fraunhofer Institute for Toxicology and Experimental Medicine, Hannover, Germany; 2 Institute of Computer Science, Goethe University Frankfurt, Frankfurt a. M., Germany

**Keywords:** Computer-aided diagnosis, Image segmentation, Neural networks, Triplet loss

## Abstract

****Purpose**:**

The integration of deep learning in image segmentation technology markedly improves the automation capabilities of medical diagnostic systems, reducing the dependence on the clinical expertise of medical professionals. However, the accuracy of image segmentation is still impacted by various interference factors encountered during image acquisition.

****Methods**:**

To address this challenge, this paper proposes a loss function designed to mine specific pixel information which dynamically changes during training process. Based on the triplet concept, this dynamic change is leveraged to drive the predicted boundaries of images closer to the real boundaries.

****Results**:**

Extensive experiments on the PH2 and ISIC2017 dermoscopy datasets validate that our proposed loss function overcomes the limitations of traditional triplet loss methods in image segmentation applications. This loss function not only enhances Jaccard indices of neural networks by 2.42$\%$ and 2.21$\%$ for PH2 and ISIC2017, respectively, but also neural networks utilizing this loss function generally surpass those that do not in terms of segmentation performance.

****Conclusion**:**

This work proposed a loss function that mined the information of specific pixels deeply without incurring additional training costs, significantly improving the automation of neural networks in image segmentation tasks. This loss function adapts to dermoscopic images of varying qualities and demonstrates higher effectiveness and robustness compared to other boundary loss functions, making it suitable for image segmentation tasks across various neural networks.

## Introduction

With the rapid advancement of computer technology, medical image analysis has evolved from a traditional dependence on the expertise of medical professionals to a method that leverages high-performance computing and sophisticated machine learning algorithms. Semantic segmentation, an indispensable component, is also the focus of this paper. Semantic segmentation uniformly assigns identical category labels to objects within the same class; for instance, different people within an image receive the same color label to denote their categorical unity [1].

Although machine learning algorithms have made significant progress in the image segmentation, factors such as image quality and object characteristics still pose challenges for accurate segmentation of object boundaries. To address these challenges, many studies have designed boundary loss functions to improve algorithm performance. Star Loss [2] encourages segmentation results to conform to a star shape by calculating the difference in predicted probabilities along the ray from the object center point to the object boundary. AC loss [3], based on active contour model, minimizes the energy inside and outside the object by determining the active contour in the image. Building on the work of [3], Euler’s elastica model was introduced in the ACE loss [4], integrating the curvature, length, and area of the active contour to enhance the connectedness of the segmented boundary. ABL [5] uses KL divergence to promote the alignment of predicted and true boundaries.

Recently, the effectiveness of triplet loss in image segmentation has been demonstrated. For available triplets, [6] enhances the convergence of the standard triplet loss by rewarding triplets that enhance the network’s convergence speed and penalizing triplets that deteriorate the network’s training ability. [7] proposed a local cross-triplet loss for discovering the similarities and differences between lesion and non-lesion regions of the breast, enabling image segmentation with small amounts of annotations.

**Fig. 1:**
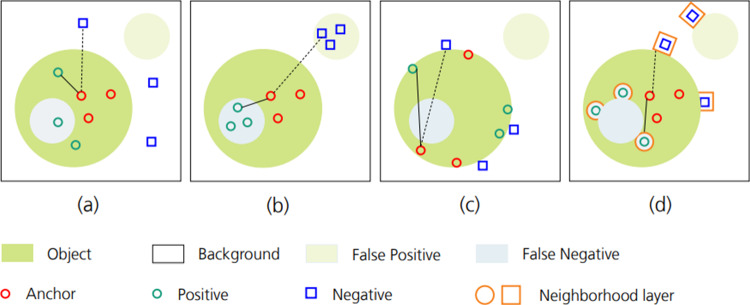
Comparative illustration of triplet selection strategies. **a** Batch all: represents positive samples with green circles (solid lines) and negative samples with blue squares (dashed lines). **b** Batch hard: incorporates the most difficult positive and negative samples. **c** Pixel-wise triplet learning: chooses samples from the boundary area of the object. **d** Adaptive neighborhood triplet selection: incorporates the neighborhood of relatively hard-to-predict samples as actual positive/negative samples

Our proposed method selects the pixel representation following neural network processing as the triplet component of the image. Regarding the selection of anchor, positive, and negative sets, two cases are considered: (i) pixels from the object form the anchor and positive sets, while pixels from the background comprise the negative set; (ii) pixels from the background serve as the anchor and positive sets, with pixels from the object constituting the negative set. To facilitate comparison of various triplet selection strategies in image segmentation, we exclusively consider case (i) in Fig. 1 at the pixel level. Batch all [8] method’s randomness in triplet selection leads to numerous triplets that meet the constraints, complicating the provision of meaningful information to the model. To address this, batch hard [8] focuses on pixels that are challenging to predict, yet this approach often results in a triplet loss that is difficult to converge, potentially hindering model convergence. Figure 1c [9] enhances the model’s boundary discriminative ability based on batch hard. However, this strategy, relying solely on boundary pixels, is not applicable to images with inhomogeneous boundary.

Inspired by boundary loss functions, this paper proposed the adaptive neighborhood triplet loss (ANTL) to address the limitations of existing triplet loss in image segmentation tasks. As shown in Fig. 1d, this method focuses on pixels and their neighborhoods that are prone to misprediction, typically located near the target boundary. The essence of our method leverages the dynamic shifts within these pixels’ neighborhoods throughout neural network training. This enables the adaptive adjustment and selection of the optimal triplet on a global scale, thereby enhancing the overall performance of the network model. In this work, the experiments applying our proposed workflow to the public datasets PH2 and ISIC2017 demonstrate significant improvements when using the neural network with ANTL, compared to baseline models. Specifically, performance on the Jaccard index is enhanced, with an increase of 2.42$\%$ and 2.21$\%$ for PH2 and ISIC2017.

## Method

### Overview of proposed image segmentation workflow

**Fig. 2:**
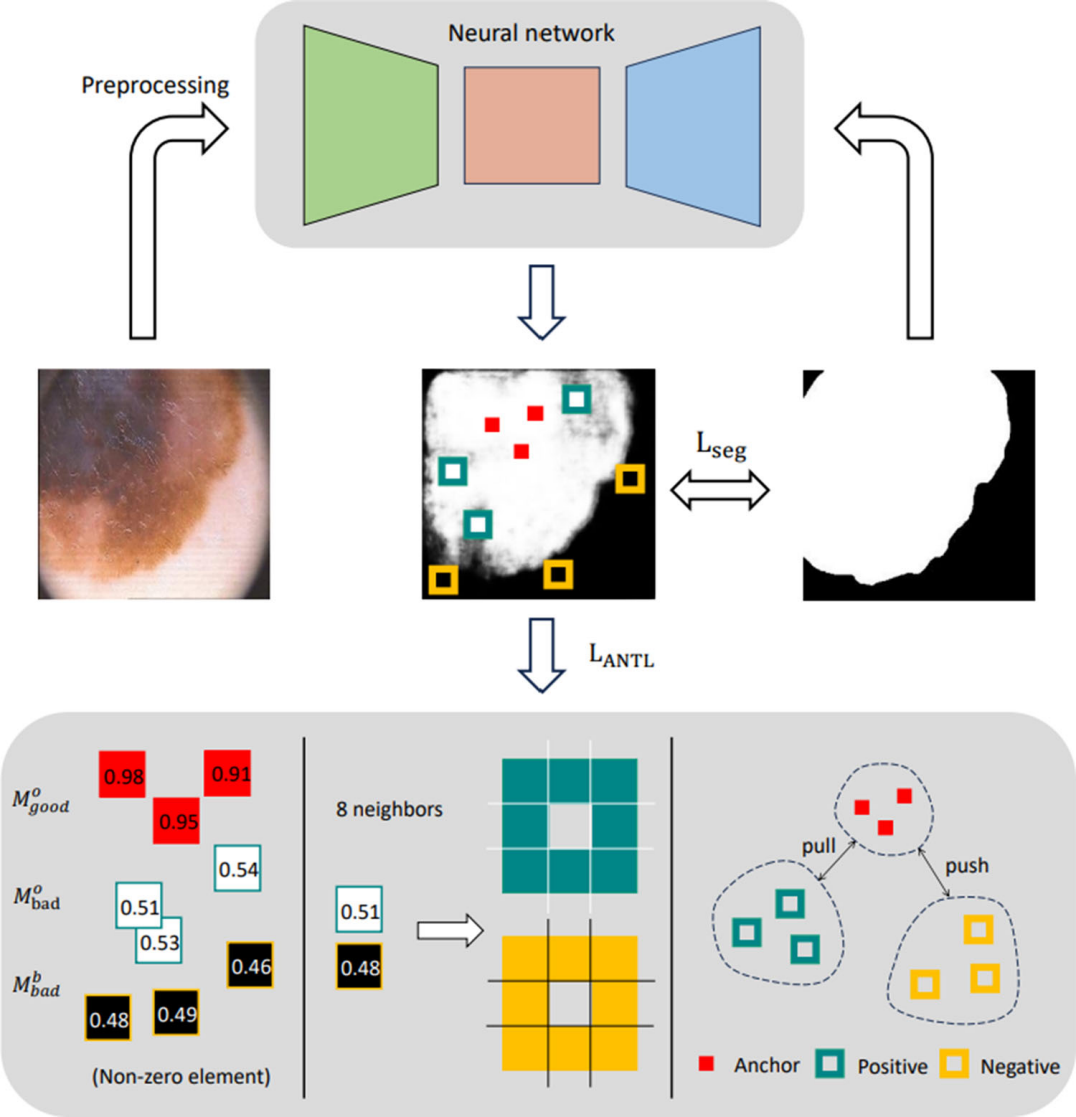
The proposed image segmentation workflow and design details. The anchor, positive, and negative components of the triplet correspond to the k-best-predicted pixels, the neighbors of k-relatively worse predicted pixels within the object, and the neighbors of k-relatively worse predicted pixels in the background, respectively (where *k* = 3)

Fig. 2 depicts the workflow of this paper, using the selection of anchors from an object as an example. The input image and its corresponding ground truth are supplied to the neural network for training and testing, following a preprocessing phase. The neural network’s output, computed from the ground truth using a softmax function, allows for the calculation of segmentation and adaptive neighborhood triplet losses ($L_{\text {seg}}$ and $L_{\text {ANTL}}$).

During the training process of the neural network, we employed various data augmentation methods, including flipping, blurring, and adjusting brightness and contrast, to enrich the dataset. The testing process of the neural network involved only resizing and normalizing the images. The neural network employs five prominent image segmentation architectures defined in segmentation models Pytorch [10]. These include Unet [11], Unet++ [12], MAnet [13], Deeplabv3+ [14] and FPN [15], each composed of an encoder, a decoder, and a segmentation head. The encoder, often referred to as the backbone network, undertakes feature extraction. We selected *ResNet 101*, pretrained on *ImageNet*, to facilitate the model’s rapid convergence. The decoder is responsible for feature fusion and the progressive categorization of each pixel. Concurrently, the segmentation head ensures the neural network’s output channel count equals the number of predicted categories. It also ensures that the size of the input image matches the size of the output predicted image by interpolation.

### Triplet loss

Assuming that $(x_i,y_i)$ represents the i-th pixel in the image and its corresponding label (object or background), respectively. The pixel $x_i$, processed by the neural network, generates a predicted probability vector $F_{\theta }(x_i)$, which contains probabilities $P^o(i)$ and $P^b(i)$ signifying whether pixel $x_i$ corresponds to the object or the background class. $\theta$ represents learnable parameters in the neural network [8, 16]. Throughout the training process of the neural network, given the existence of the *i*-th, *j*-th, and *k*-th pixels in the image, their corresponding labels comply with the condition $y_i^a=y_j^p\ne y_k^n$ [17], which can be amalgamated into the triplet $T=(x_i^a,x_j^p,x_k^n)$. Herein, the superscripts a, p, and n signify the anchor, positive, and negative components of the triplet, respectively.

The triplet loss function reduces the difference within the anchor-positive pair ($x_i^a$, $x_j^p$), bringing pixels of the same category closer in predicted probabilities, while expanding the difference within the anchor-negative pair ($x_i^a$, $x_k^n$) to separate pixels from different categories. This difference in predicted probabilities can be transformed into a Euclidean distance between two vectors. In the ideal scenario, where the pixels within the triplet are accurately predicted by the neural network, denoted as $F_{\theta }(x_i)=y_i$, define $\Delta$ as the difference between the Euclidean distances within the anchor-positive pair and the anchor-negative pair. Consider a set of triplets as an example, the Euclidean distance difference between the anchor-positive and anchor-negative pairs is calculated. The discrepancy between this difference and $\Delta$ is utilized as the loss term, expressed as follows,

1 $$\begin{aligned} l = [||F_{\theta }(x_i^a)-F_{\theta }(x_j^p)||_2^2-||F_{\theta }(x_i^a)-F_{\theta }(x_k^n)||_2^2+\Delta ] \nonumber \\ \end{aligned}$$

$||\cdot ||_2^2$ denotes the euclidean distance between two vectors.

### Triplet selection

Building on the discussion in Section “Introduction” regarding triplet selection, we examine the neighborhood layer of relatively hard-to-predict pixels to segment within both the object and the background, which will be abbreviated as *o* and *b*. By setting the number of triplets to k, the proposed ANTL is computed through the following three principal steps.

#### Selection of k-best and k-relatively worse predicted pixels within the same labeled region

For any pixel in a two-dimensional image with width *w* and height *h*, the corresponding coordinate (*m*, *n*) has a value *x*(*m*, *n*) and a label *y*(*m*, *n*). The prediction probability obtained from the neural network, which contains ${\theta }$ trainable parameters, is $F_{\theta }{(x(m,n))}=(P^o_{\text {init}}(m,n),P^b_{\text {init}}(m,n))$. Thus, for all $m \in \{1,...,w\}$ and $n \in \{1,...,h\}$, $P^o_{\text {init}}$ and $P^b_{\text {init}}$ denote the predicted probabilities of pixels being labeled as object and background, respectively, represented in the form of two-dimensional matrices with the size $w \times h$. The indicator function, denoted as $\mathbbm {1}_{\text {condition}}$, outputs 1 when the condition is true and 0 when it is false. For $class \in \{o, b\}$, there is the following general representation,

2 $$\begin{aligned} P^{\text {class}}(m,n)=P^{\text {class}}_{\text {init}}(m,n) \cdot \mathbbm {1}_{y(m,n)=\text {class}} \end{aligned}$$

The new matrix $P^{\text {class}}$, derived from the initial matrix $P^{\text {class}}_{\text {init}}$ by applying the indicator function, represents the predicted probability of the pixel belonging to the current class when its label matches the current class; otherwise, the output is 0.

The nonzero elements in matrices $P^o$ and $P^b$ are arranged in ascending order, and the predicted probabilities corresponding to the elements in positions $(w \cdot h-(k+1))$ are defined as $p_{-(k+1)}^o$ and $p_{-(k+1)}^b$, respectively. For each of the two classes (*o*, *b*), we define a matrix $M_{\text {good}}^{\text {class}}$. This matrix is satisfied for all $m \in \{1,...,w\}$ and $n \in \{1,...,h\}$:

3 $$\begin{aligned} M_{\text {good}}^{\text {class}}(m,n)=P^{\text {class}}(m,n) \cdot \mathbbm {1}_{P^{\text {class}}(m,n)>p_{-(k+1)}^{\text {class}}} \end{aligned}$$

thereby filtering the *k* predicted best pixels in the region labeled as object and background, respectively.

We characterize relatively hard-to-predict pixels as those with a high likelihood of being misclassified. For example, a pixel belonging to the object class might have a predicted probability of 0.51 for object class and 0.49 for background class. Let $(l^{\text {class}}, u^{\text {class}})$ represent the search interval on $P^{\text {class}}$ and set initial search interval to (0.5,0.55). For each of the two classes, we define a matrix $M_{\text {bad}}^{\text {class}}$. This matrix is satisfied for all $m \in \{1,...,w\}$ and $n \in \{1,...,h\}$:

4 $$\begin{aligned} M_{\text {bad}}^{\text {class}}(m,n)=P^{\text {class}}(m,n) \cdot \mathbbm {1}_{l^{\text {class}}<P^{\text {class}}(m,n)<u^{\text {class}}} \end{aligned}$$

After completing one search round, if the number of nonzero elements in $M_{\text {bad}}^{\text {class}}$ remains below k, $u^{\text {class}}$ is increased by 0.05, initiating another search round. This process continues until the number of nonzero elements in $M_{\text {bad}}^{\text {class}}$ reaches or exceeds k. In scenarios where all pixels belonging to a certain class are incorrectly predicted ($\forall P^{\text {class}}(m,n) \in P^{\text {class}}, P^{\text {class}}(m,n) < 0.5$), $l^{\text {class}}$ is decreased by 0.05. A new search begins and continues in a similar manner until the number of nonzero elements in $M_{\text {bad}}^{\text {class}}$ is greater than or equal to k.

Following the aforementioned procedure, if the count of nonzero elements in $M_{\text {bad}}^{\text {class}}$ exceeds k, a random selection of k elements is made from all nonzero elements. The elements that are not selected are set to zero.

Matrices $M_{\text {good}}^{\text {class}}$ record the k-best-predicted pixels in the object and background regions. These matrices encompass not only the predicted values of the respective pixels but also contain location information within the image. Similarly, $M_{\text {bad}}^{\text {class}}$ is dedicated to identifying the *k* pixels that are relatively hard to predict. Pixels that do not satisfy the conditions are assigned zero values in the matrix.

#### Identifying the neighborhood of nonzero elements in $M_{\text {bad}}^{\text {class}}$

Last section selects the k-best and k-relatively worse predicted pixels within the same labeled region, and their corresponding predictions are recorded in the matrices $M_{\text {good}}^{\text {class}}$ and $M_{\text {bad}}^{\text {class}}$. This section focuses on collecting the neighborhood of k-relatively worse predicted pixels (nonzero elements in $M_{\text {bad}}^{\text {class}}$), and recording their corresponding predictions in the matrix $N_{\text {bad}}^{\text {class}}$.

In a two-dimensional image, we consider a pixel located at the coordinate (*m*, *n*). The eight neighbors of the pixel are positioned at coordinates $(m-1,n-1)$, $(m-1,n)$, $(m-1,n+1)$, $(m,n-1)$, $(m,n+1)$, $(m+1,n-1)$, $(m+1,n)$, and $(m+1,n+1)$, representing the vertical and horizontal neighbors that form its surrounding region. When the neighbors are identified, their respective predicted probabilities are collected by the two-dimensional matrix $N_{\text {bad}}^{\text {class}}$, which is defined over a $w \times h$ space. This process ensures that neighbors in the image located at identical coordinates are not counted multiple times. Assuming that the *r*-th nonzero element is located at $(m_r,n_r)$, where $r \in \{1,...,k\}$, its eight neighbors can be expressed as,

5 $$\begin{aligned} n_8(m_r,n_r){} & {} =\{(m_r+r_1,n_r+r_2)|\nonumber \\{} & {} \quad -1\le r_1,r_2 \le 1, (r_1,r_2)\ne (0,0)\} \nonumber \\ \end{aligned}$$

and satisfy condition $0\le m_r+r_1<w, 0\le n_r+r_2 <h$, without exceeding the image boundary. Thus, for all $(m_r+r_1,n_r+r_2)\in n_8$,

6 $$\begin{aligned} \begin{aligned} N_{\text {bad}}^{\text {class}}(m_r+r_1,n_r+r_2)=P^{\text {class}}(m_r+r_1,n_r+r_2) \end{aligned} \end{aligned}$$

Utilizing equation 2, the label corresponding to nonzero element at $(m_r,n_r)$ must match the labels of its eight neighbors. Neighbors with non-matching labels are excluded from $N_{\text {bad}}^{\text {class}}$.

To more effectively analyze the impact of neighboring regions on neural network performance, the scope of these neighboring regions is expanded. Employing Eq. 6, we define $N_{\text {bad}^1}^{\text {class}}(m_r+r_1,n_r+r_2)$ as the first neighboring layer at pixel $(m_r,n_r)$. If Eq. 5 is applied to each nonzero element in matrix $N_{bad^1}^{class}$, it results in the identification of $N_{\text {bad}^2}^{\text {class}}$, which represents the second neighboring layer for each nonzero element. Following this methodology, the *i*-th neighboring layer can be identified as $N_{\text {bad}^i}^{\text {class}}$. The $N_{\text {bad}^i}^{\text {class}}$ matrix was introduced to substitute the nonzero elements in $M_{\text {bad}}^{\text {class}}$ with neighboring layer information for inclusion in the loss function calculation. As training progresses, this neighboring layer information increasingly reflects the model’s performance more accurately compared to the nonzero elements in $M_{\text {bad}}^{\text {class}}$.

#### Computing the ANTL

Our proposed loss term is separated into two situations, based on the anchors derived from the object and background region. When the k-best-predicted pixels in the object region serve as anchors, the neighboring layer corresponding to the *k* relatively hard-to-predict pixels within the same object region is labeled positive. Conversely, the neighboring layer corresponding to the *k* relatively hard-to-predict pixels in the background region is labeled negative. Employing the *k* best-predicted pixels in the background region as anchors similarly results in analogous triplets. Integrating step 1 and step 2, the triplet comprises nonzero elements in $M_{\text {good}}^{c1}$, $N_{\text {bad}}^{c1}$ and $N_{\text {bad}}^{c2}$, under the condition that $c1\in \{o, b\}$ and $c2\in \{b, o\}$. In computing the triplet, it satisfies,

7 $$\begin{aligned} l_{\text {antl}}^{c1}= & {} \left[ \left\| \frac{1}{k}\sum _{i1=1}^kM_{\text {good}}^{c1}(m_{i1},n_{i1})-\frac{1}{p}\sum _{i2=1}^pN_{\text {bad}}^{c1}(m_{i2},n_{i2})\right\| _2^2\right. \nonumber \\{} & {} \left. -\left\| \frac{1}{k}\sum _{i1=1}^kM_{\text {good}}^{c1}(m_{i1},n_{i1})-\frac{1}{q}\sum _{i3=1}^qN_{\text {bad}}^{c2}(m_{i3},n_{i3})\right\| _2^2 +\Delta \right] \nonumber \\ \end{aligned}$$

where *k*, *p*, and *q* denote the number of nonzero elements in the $M_{\text {good}}^o$, $N_{\text {bad}}^o$, and $N_{bad}^b$, respectively.

Pixels belong to object and background regions are labeled as 1 and 0. Ideally, when pixels in the object region are chosen as anchors and the relevant pixels are all correctly predicted, the values of expressions satisfy $M_{\text {good}}^o(m_{i1},n_{i1}) = N_{\text {bad}}^o(m_{i2},n_{i2})=1$, while $N_{bad}^b(m_{i3},n_{i3})=0$. Similarly, when pixels in the background region are chosen as anchors and the relevant pixels are all correctly predicted, the values of expressions satisfy $M_{\text {good}}^b(m_{j1},n_{j1})=N_{\text {bad}}^b(m_{j2},n_{j2})=0$, while $N_{\text {bad}}^o(m_{j3},n_{j3})=1$. In these ideal scenarios, since the loss term is 0, the corresponding $\Delta$ values will be 1.

### Overall loss function

Regarding image segmentation, we additionally employ the classical cross-entropy loss function, which is defined as,

8 $$\begin{aligned} L_{\text {ce}}=-\frac{1}{N}\sum _{x}[g\,lnS+(1-g)\,ln(1-S)] \end{aligned}$$

while *x* stands for the sample, *g* signifies the actual value and *S* corresponds to the output of the model after softmax function. *N* denotes the total number of samples. The objective function, based on Eqs. 7 and 8, is defined as follows:

9 $$\begin{aligned} L=\alpha (L_{\text {antl}}^o+L_{\text {antl}}^b)+L_{\text {ce}} \end{aligned}$$

To maintain a balance between the ANTL and cross-entropy loss, we set the weight of the former at $\alpha$. The selection of $\alpha$ will be discussed in subsequent sections.

## Experimental results

### Experiment setup

In this study, we evaluate model performance by using two public dermoscopy datasets, PH2 [18] and ISIC2017 [19]. For $512 \times 512$ images, batch sizes were configured as 4 and 16 for PH2 and ISIC2017, with early stopping mechanisms set at 10 and 16 epochs, respectively. If no performance improvement is observed on the validation set over 8 consecutive epochs during training, the learning rate is reduced by half. We selected SGD as the optimizer, with an initial learning rate of 0.1. To enhance the reliability of our experimental results, we utilized three distinct random seeds in all experiments. All experiments were performed on the DGX A100 server equipped with 8 Nvidia A100 Tensor Core GPUs (total memory 320GB).

### Comparative analysis of image segmentation across multiple datasets

To explore the effectiveness of the proposed ANTL, this study compares the segmentation performance of boundary loss functions, including Starloss [2], AC [3], ACE [4], and ABL [5], on the PH2 and ISIC2017 datasets. Furthermore, we extend the comparison of these loss functions to five principal image segmentation architectures: Unet, Unet++, MAnet, Deeplabv3+ and FPN. The Jaccard index which ranges from 0(worst) - 100(best), as recommended by the ISIC challenge, is adopted as the performance metric.

**Table 1:** Quantitative segmentation results (mean ± standard deviation) of five neural networks on the PH2 dataset

Neural Networks	Methods	Common	Atypical	Melanoma	Sum
Unet	Baseline	88.06 ± 1.03	87.08 ± 3.92	74.43 ± 1.49	84.94 ± 2.13
	Starloss	**90**.**96** ± 0.85	86.38 ± 0.31	74.46 ± 1.13	85.83 ± 0.48
	AC	89.72 ± 0.53	88.57 ± 0.44	72.07 ± 0.86	85.73 ± 0.32
	ACE	89.24 ± 2.39	87.50 ± 1.97	72.31 ± 1.27	85.16 ± 1.89
	ABL	89.39 ± 0.77	85.30 ± 0.96	71.63 ± 1.56	84.20 ± 0.51
	ANTL	90.27 ± 0.14	**89.00 ± 2.35**	**77.20 ± 0.75**	**87.15 ± 1.11**
Unet++	Baseline	91.03 ± 0.17	86.46 ± 0.30	71.10 ± 1.87	85.22 ± 0.67
	Starloss	90.63 ± 0.45	86.45 ± 0.23	72.56 ± 0.84	85.34 ± 0.16
	AC	89.46 ± 0.45	86.51 ± 0.47	73.07 ± 1.13	85.00 ± 0.23
	ACE	90.13 ± 0.40	**87.37 ± 0.67**	**74.98 ± 1.45**	85.99 ± 0.68
	ABL	90.01 ± 0.42	87.19 ± 0.91	73.81 ± 1.45	85.65 ± 0.78
	ANTL	**91.88 ± 0.29**	87.15 ± 0.97	72.67 ± 1.09	**86.14 ± 0.65**
MAnet	Baseline	88.21 ± 1.41	84.29 ± 0.82	**76.64 ± 0.81**	84.33 ± 0.57
	Starloss	87.42 ± 1.55	83.65 ± 0.30	73.85 ± 3.46	83.20 ± 0.27
	AC	87.40 ± 1.46	85.19 ± 1.78	72.86 ± 2.49	83.61 ± 0.49
	ACE	87.35 ± 1.60	86.25 ± 2.32	73.52 ± 1.58	84.14 ± 1.69
	ABL	88.01 ± 0.68	85.43 ± 2.45	74.48 ± 2.07	84.27 ± 1.63
	ANTL	**90.09 ± 0.95**	**88.05 ± 1.49**	74.43 ± 1.62	**86.14 ± 1.13**
Deeplabv3+	Baseline	88.78 ± 0.74	84.48 ± 1.07	76.71 ± 2.07	84.64 ± 0.23
	Starloss	90.17 ± 1.18	84.50 ± 0.76	74.46 ± 2.97	84.76 ± 1.18
	AC	87.80 ± 1.71	83.20 ± 0.99	74.03 ± 2.04	83.20 ± 1.40
	ACE	89.95 ± 0.87	84.77 ± 0.28	74.14 ± 0.99	84.72 ± 0.49
	ABL	90.28 ± 0.75	86.45 ± 2.76	75.52 ± 3.34	85.80 ± 1.48
	ANTL	**90.90 ± 0.28**	**86.61 ± 0.86**	**79.33 ± 1.08**	**86.87 ± 0.05**
FPN	Baseline	87.65 ± 0.96	84.40 ± 1.40	71.38 ± 1.07	83.10 ± 0.21
	Starloss	87.91 ± 0.95	86.64 ± 1.10	71.66 ± 2.21	84.16 ± 0.09
	AC	87.69 ± 0.51	**87.30 ± 1.11**	69.69 ± 2.41	83.94 ± 0.92
	ACE	88.24 ± 0.91	86.12 ± 2.55	73.41 ± 2.16	84.42 ± 1.29
	ABL	86.52 ± 2.76	85.53 ± 2.75	70.39 ± 6.69	82.90 ± 0.74
	ANTL	**88.25 ± 1.16**	87.09 ± 1.29	**76.92 ± 2.73**	**85.52 ± 1.21**

Under the same training settings, the best segmentation results are in bold.

The experiments of each neural network include Starloss, AC, ACE, ABL, and our proposed ANTL on the three categories of the test set (common, atypical, melanoma) and the entire test set (sum)

**Table 2:** Quantitative segmentation results (mean  ±  standard deviation) of five neural networks on the ISIC2017 dataset

Neural networks	Methods	Nevus	Seborrheic keratosis	Melanoma	Sum
Unet	Baseline	77.82 ± 0.30	64.15 ± 0.50	66.70 ± 0.57	73.60 ± 0.32
	AC	77.51 ± 0.40	63.45 ± 0.37	67.22 ± 0.07	73.39 ± 0.36
	ACE	77.55 ± 0.28	63.42 ± 0.80	67.30 ± 0.20	73.44 ± 0.34
	ABL	77.21 ± 0.65	64.61 ± 0.65	67.15 ± 0.32	73.36 ± 0.57
	ANTL	**78.49 ± 0.60**	**66.85 ± 0.37**	**68.65 ± 1.48**	**74.83 ± 0.67**
Unet++	Baseline	77.23 ± 1.14	64.21 ± 0.69	67.39 ± 0.31	73.36 ± 0.78
	AC	77.84 ± 0.30	63.94 ± 0.54	67.70 ± 0.07	73.78 ± 0.17
	ACE	78.23 ± 0.14	65.20 ± 0.80	67.99 ± 0.61	74.28 ± 0.15
	ABL	77.86 ± 0.21	65.13 ± 0.85	67.48 ± 0.67	73.93 ± 0.39
	ANTL	**79.35 ± 0.34**	**66.31 ± 1.45**	**68.56 ± 0.69**	**75.29 ± 0.54**
MAnet	Baseline	77.89 ± 0.92	65.26 ± 1.77	67.98 ± 1.21	74.06 ± 0.95
	AC	77.53 ± 0.27	64.86 ± 0.44	67.61 ± 0.37	73.69 ± 0.27
	ACE	78.23 ± 0.62	65.90 ± 1.29	67.52 ± 0.45	74.29 ± 0.67
	ABL	76.93 ± 0.81	62.94 ± 0.87	66.60 ± 1.60	72.82 ± 0.90
	ANTL	**80.03 ± 1.00**	**67.96 ± 1.07**	**70.04 ± 0.42**	**76.27 ± 0.77**
Deeplabv3+	Baseline	78.49 ± 0.42	64.67 ± 0.36	68.02 ± 0.58	74.38 ± 0.40
	AC	78.57 ± 0.32	64.19 ± 0.50	68.27 ± 0.27	74.40 ± 0.31
	ACE	78.67 ± 0.59	**65.50 ± 0.53**	**68.60 ± 0.62**	74.73 ± 0.21
	ABL	78.46 ± 0.37	64.71 ± 0.84	67.98 ± 0.51	74.35 ± 0.43
	ANTL	**79.55 ± 0.06**	65.48 ± 0.71	**68.60 ± 0.09**	**75.30 ± 0.16**
FPN	Baseline	77.24 ± 0.60	64.96 ± 0.82	68.17 ± 1.28	73.63 ± 0.52
	AC	77.76 ± 0.15	64.91 ± 0.62	67.76 ± 0.57	73.88 ± 0.29
	ACE	77.53 ± 0.18	64.79 ± 0.78	67.58 ± 0.62	73.68 ± 0.33
	ABL	78.12 ± 0.33	64.07 ± 0.90	67.73 ± 0.63	73.98 ± 0.45
	ANTL	**79.19 ± 0.58**	**66.48 ± 0.50**	**68.27 ± 0.64**	**75.15 ± 0.58**

Under the same training settings, the best segmentation results are in bold.

The experiments of each neural network include AC, ACE, ABL, and our proposed ANTL on the three categories of the test set (nevus, seborrheic keratosis, melanoma) and the entire test set (sum)

According to the experimental results shown in Table 1 and Table 2, the proposed ANTL achieved better results than other boundary loss functions across five neural networks. Specifically, on the PH2 dataset, the FPN architecture records a maximum performance improvement of 2.42$\%$. On the ISIC2017 dataset, MAnet shows a maximum improvement of 2.21$\%$. Although the neural networks incorporating ANTL did not achieve the best segmentation results in all categories, their overall advantage across various datasets and neural networks demonstrates the effectiveness and robustness of the method. It should be noted that due to the long training time of Starloss on ISIC2017, we could not include it in Table 2 (for example, Unet took an average of 9–11 min per epoch with other loss functions, whereas Starloss took 5 h and 58 min to complete one epoch).

**Fig. 3:**
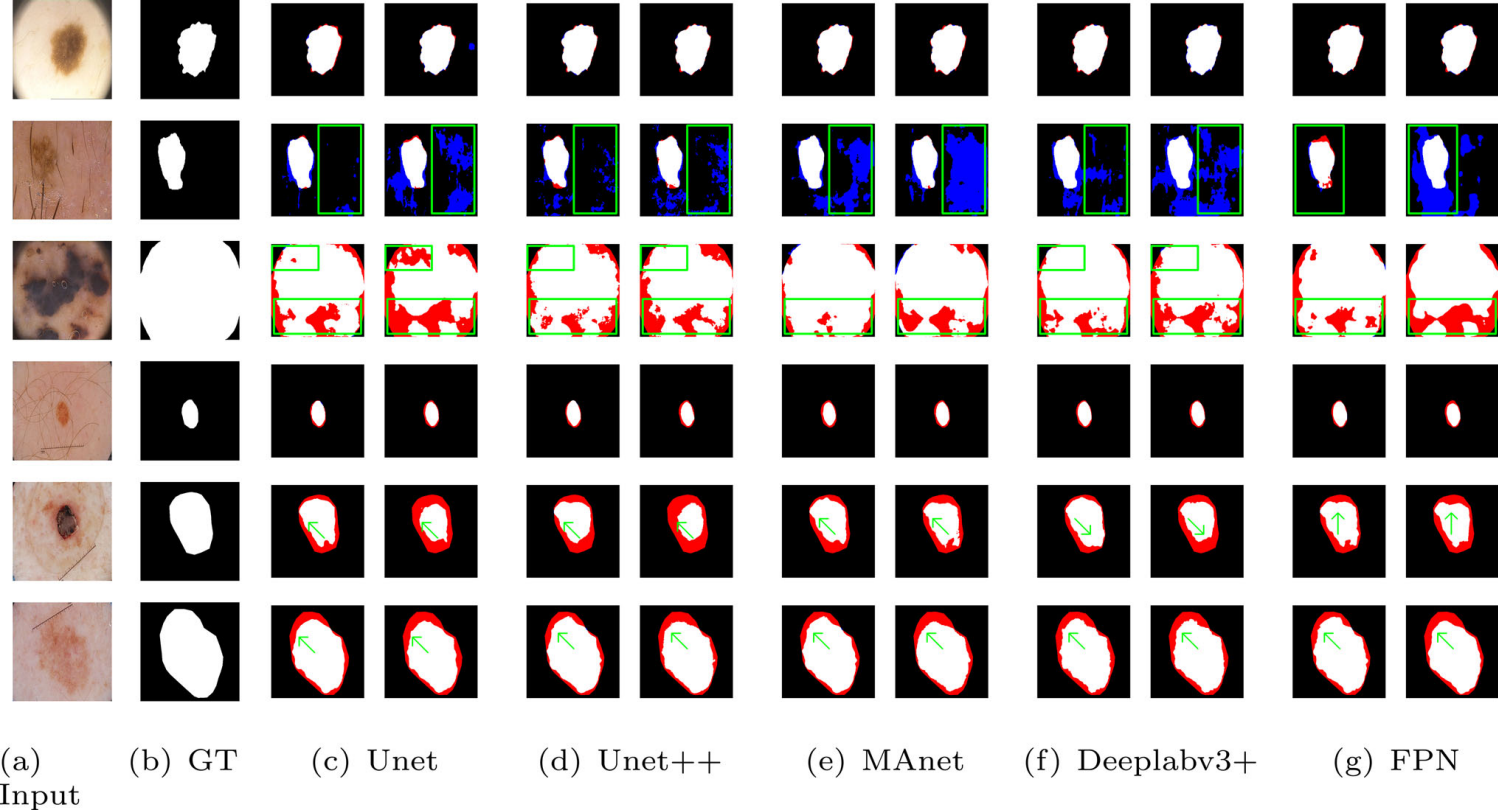
Segmentation results from different neural networks for common nevi, atypical nevi and melanoma (from 1st-3rd row) in the PH2 testset and for nevus, seborrheic keratosis and melanoma (from 4th-6th row) in the ISIC2017 testset (Red: false negatives; Blue: false positives). **a** and **b** display the input image and ground truth (GT). The left and right columns from **c** to **g** depict qualitative results with/without ANTL

Furthermore, to comprehensively analyze the model’s image segmentation capabilities across different datasets, Fig. 3 illustrates the segmentation results within the PH2 (containing common nevi, atypical nevi and melanoma) and ISIC2017 (containing nevus, seborrheic keratosis and melanoma) datasets, with and without integrating ANTL. For image categories with high contrast and distinct boundaries between the object and background, such as common nevi and nevus, both the baseline and the enhanced model with ANTL perform effectively in segmentation tasks. ANTL demonstrates a subtle advantage at the boundaries of the correctly segmented regions. This advantage is reflected that the Jaccard index of the neural network with ANTL increased by up to 2.21$\%$ in the common nevi class of PH2 and 2.14$\%$ in the nevus class of ISIC2017.

This advantage is also evident when processing images with blurred boundaries and low contrast, such as atypical nevi, seborrheic keratosis, and melanoma. Specifically, the model with ANTL facilitates a more efficient advancement of the correct segmentation region’s boundary (white) into the incorrect segmentation regions (red/blue). This efficiency is manifest in the closer approximation of the predicted boundary to the GT boundary, as indicated by the green arrow. In the PH2 dataset, the Jaccard Index for atypical nevi and melanoma increased by up to 3.76$\%$ and 5.54$\%$, respectively. In the ISIC2017 dataset, the Jaccard Index for seborrheic keratosis and melanoma increased by up to 2.70$\%$ and 2.06$\%$, respectively.

**Fig. 4:**
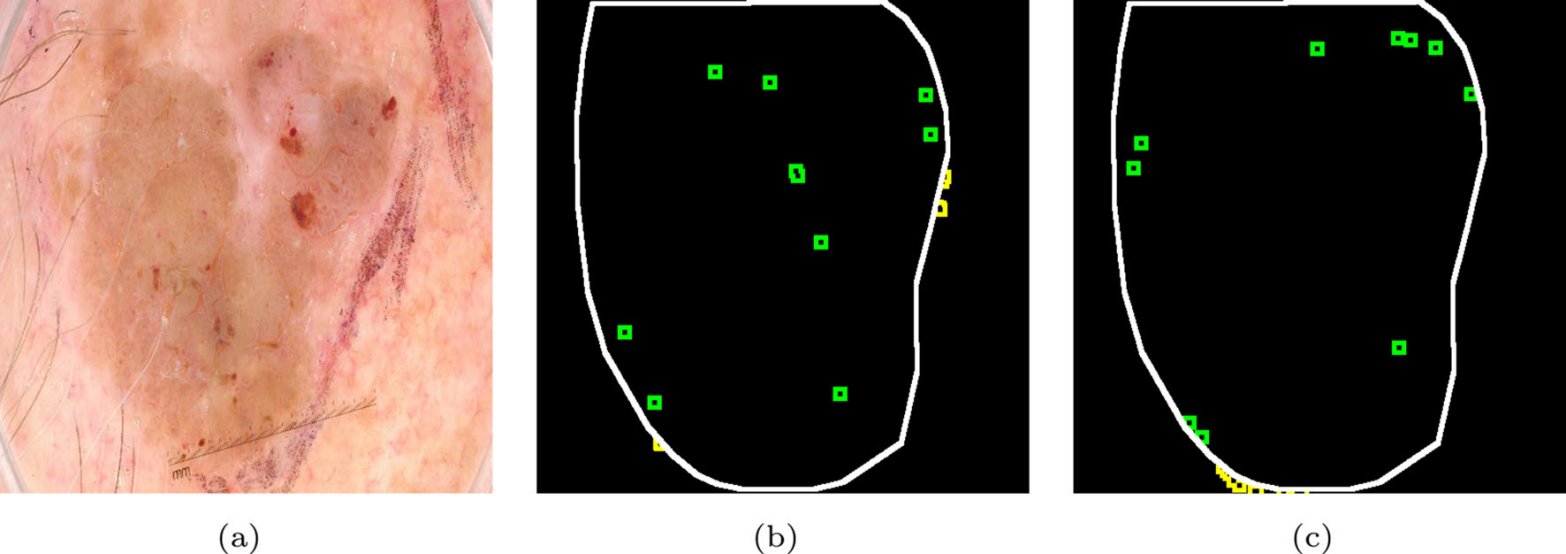
Distribution of relatively hard-to-segment pixels in ISIC2017 across various training stages. **a** Original image; **b** early training stage; **c** Late training stage. White: segmented boundary of GT; green: neighboring region of hard-to-segment pixels in the object; yellow: neighboring region of hard-to-segment pixels in the background

Figure 4 illustrates the changing positions of relatively hard-to-segment pixels in the image at different training stages, using pixels within the object as anchors. In the early training stage, the distribution of these relatively hard-to-segment pixels across the image exhibits irregularity. Due to the poor predictive performance of the neural network at this stage, it is possible that the specified k number of pixels may not be found. In the object region, ANTL prompts neighboring regions (positive) of these pixels to be closer with the anchor predictions. Conversely, in the background region, ANTL penalizes neighboring regions (negative) of these pixels by shifting their predictions away from the predictions of the anchors. As training progresses, pixels that are hard to segment gradually cluster near the predicted boundary of the object. This trend in the predictions encourages neighboring regions located at the predicted boundary to converge toward the pixels that have been accurately segmented, effectively pushing the predicted boundary progressively closer to the true boundary.

### Further analysis in ANTL

**Fig. 5:**
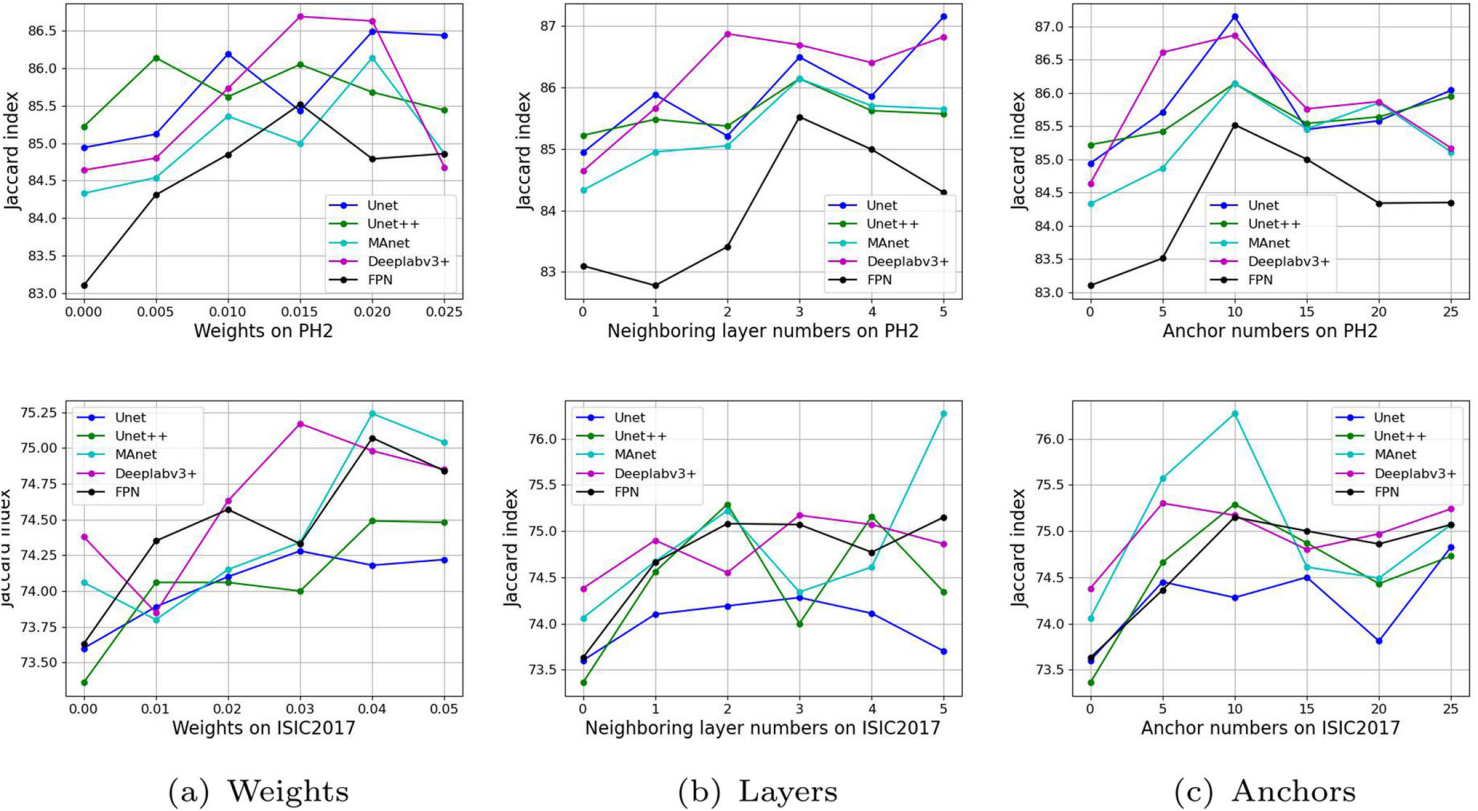
Variations in weight, number of neighboring layers, and anchor effects on the Jaccard index for U-Net, U-Net++, MAnet, DeepLabv3+, and FPN

In this section, we adjust three parameters in the ANTL: weight, number of neighboring layers and anchors, to select the optimal settings for the performance comparison in Section "Comparisontrl". Figure 5 illustrates that the optimal parameters, weight, number of neighboring layers and anchors, differ across various datasets and neural networks.

It is critical to appropriately set the weights to balance the two loss functions when integrating the new loss function into the existing image segmentation learning framework. The PH2 and ISIC2017 datasets reached their highest Jaccard index values with weights of 0.015 and 0.04, respectively. Furthermore, the experiments in Fig. 5a also reveal that excessively low ANTL weights can disrupt the training process in some networks, resulting in their performance degradation. Figure 5b illustrates the impact of the number of neighboring layers on the performance of models, specifically for pixels that are relatively hard to predict. The optimal performances on the PH2 and ISIC2017 datasets which are from Unet and MAnet are achieved when the computation of the ANTL spans the 1st to the 5th neighboring layers, respectively. We evaluated 5 anchor configurations (5, 10, 15, 20, and 25), as depicted in Fig. 5c. Analysis of these configurations reveals that increasing the number of anchors generally improves model performance. Specifically, setting the number of anchors to 10 yielded the best results: the Unet model achieved its highest Jaccard index on the PH2 dataset, and the MAnet model on the ISIC2017 dataset.

Drawing on these experimental findings, we established specific weights, numbers of neighboring layers and anchors for implementing the ANTL across five neural networks, thereby refining the experimental outcomes presented in Section “Comparisontrl”.

## Conclusion

The ANTL, as proposed in this paper, can be efficiently integrated into the image segmentation workflow without incurring additional training costs. This workflow automates the entire process, from image preprocessing to model performance evaluation. By adaptively selecting the neighborhoods of pixels that are relatively difficult to predict in images, the neural network that incorporates this loss function enhances its ability to differentiate image details. Furthermore, this loss function effectively addresses the issues of model instability and gradient explosion that are often encountered with classical triplet losses in image segmentation tasks. It is also adaptable, being applicable to images of various types and with differing boundary qualities. Experimental results on the public dermatoscopy datasets PH2 and ISIC2017 show that the ANTL is not only more robust than other boundary losses but also demonstrates superior image segmentation performance across five different neural network architectures.

In future studies, we aim to apply the proposed loss function to more diverse datasets. This will enable us to further validate its robustness and explore its potential applications in other image analysis tasks.

## A implementation details

This section supplements Section “Workflow” by detailing the experimental setup for the PH2 and ISIC2017 datasets within our workflow.

Since the PH2 dataset does not include an official split, we divided it into training, validation, and test sets using a 70$\%$, 10$\%$, and 20$\%$ split, respectively. To ensure an even distribution of the three categories (common nevi, atypical nevi, and melanoma) across the sets, we divided the images within each category according to the above proportions. The ISIC2017 dataset, which provides official training, validation, and test sets, was used directly in this study. Tables 3 and 4 show the distribution of images across the training, validation, and test sets for the PH2 and ISIC2017 datasets, respectively.

The image sizes in the PH2 dataset are same at 767$\times$576 pixels, while the ISIC dataset image sizes range from 639$\times$602 to 6768$\times$4449 pixels. Considering the neural network’s requirement for consistent input image sizes and the computational capacity of our local devices, we resized all images to 512$\times$512 pixels during the data preprocessing stage. Additionally, we applied various data augmentation techniques to the training sets of both datasets to enhance their quality (see Section "Workflow" for details).

The roles of the training, validation and test sets in this paper are similar to those of previous work: The training set is used to train the neural network; the validation set is used for tuning and selecting the best model; and the test set is only used for the performance evaluation of the final model and is not involved in the training and validating process. Table 1 and 2 show the scores of the test set on the final model.

**Table 3:** Label distribution in PH2 dataset

Class name	Train count	Validation count	Test count
Common nevi	56	16	8
Atypical nevi	56	16	8
Melanoma	28	8	4

**Table 4:** Label distribution in ISIC2017 dataset

Class came	Train count	Validation count	Test count
Nevus	1372	78	393
Seborrheic keratosis	254	42	90
Melanoma	374	30	117
